# Design and testing of a posture-adjusting precision metering device for high-speed maize planting

**DOI:** 10.3389/fpls.2025.1458597

**Published:** 2025-01-27

**Authors:** Jianxin Dong, Xiaojun Gao, Zhouzhou Zheng, Pengfei Zhao, Yubin Bi, Yuxiang Huang

**Affiliations:** ^1^ College of Mechanical and Electronic Engineering, Northwest A&F University, Xianyang, China; ^2^ Scientific Research Base for Conservation Tillage Mechanization (Yellow River Basin) of Ministry of Agriculture and Rural Affairs, Northwest A&F University, Xianyang, China; ^3^ Shaanxi Engineering Research Center for Agricultural Equipment, Northwest A&F University, Xianyang, China

**Keywords:** maize, seed-metering device, posture adjustment, seed flow, high-speed planting

## Abstract

**Introduction:**

This study introduces a new technical method that involves adjusting the seed-filling posture and fluidizing the seed group to improve the capacity of mechanical seed-metering devices for high-speed maize planting. The design of a novel posture-adjusting seed-metering device is presented and its operating principle is described. In the seed-filling area, the seeds maintain the same postural characteristics and are detached from the seed group. This method creates favorable conditions for mechanical seed-metering devices to achieve effective seed-filling under high-speed planting conditions.

**Methods:**

Firstly, the key parameters of the core component were analyzed. Secondly, the influences of the seed posture adjusting efficiency and seed flow distribution characteristics on the seed-filling effect were clarified by the EDEM simulation. Finally, the high-speed metering performance was optimized and validated using bench testing.

**Results:**

The experiment demonstrated that the type A groove had the highest seed posture adjusting efficiency, a more uniform seed flow distribution, and a superior seed-filling effect as compared to the other groove types. The best metering performance was achieved with an opening angle of 21.6° and an opening width of 8.4 mm; this resulted in pass, repeat, and leak rates of 94.8%, 1.3%, and 3.9%, respectively. Within the speed range of 8–14 km/h, the posture-adjusting seed-metering device demonstrated a pass rate of over 94%, a repeat rate below 2.1%, and a leak rate below 3.9%.

**Discussion:**

The designed device had a better high-speed planting capacity than a seed-metering device without a posture-adjusting mechanism, thus proving the effectiveness of the novel seed-filling method. These findings provide a reference for improving the high-speed planting capacity of mechanical seed-metering devices.

## 1 Introduction

The consumption of maize, a widely grown food crop worldwide, is increasing yearly (Hao et al., 2017; Cay et al., 2018). While continued expansion of the scale of agricultural production is needed, crop yields and production efficiency also require rapid improvement. A planter is required to achieve dense and precise planting functions. Improvements in maize planting technology are needed to develop higher-speed and more precise planting devices (Yang et al., 2016a; Kostić et al., 2018; Virk et al., 2019).

Seed-metering devices offer precise seed-planting technology, and their performance directly influences the seeding quality (Yang et al., 2015, 2016b). Mechanical seed-metering devices are extensively used in maize production due to their reliable planting capacity at low and medium speeds as well as their cost-effectiveness, compatibility, and simple structures (Wang et al., 2017a; Xue et al., 2019; Shen et al., 2021). However, the reliability of mechanical seed-metering devices substantially reduces with increases in planter speed. These devices cannot satisfy the demand for high-speed (over 8 km/h) precision seeding, which limits their practical application (Jia et al., 2018; Wang et al., 2019; Yan et al., 2020; Huang et al., 2022). This is primarily because mechanical seed-metering devices are not adaptable to the intricate shapes of seeds. Continuously and successfully filling seeds during high-speed planting is challenging, resulting in repeated or leaky seeding, which significantly influences the maize yield and quality (Li et al., 2023a; Geng et al., 2016; Liu et al., 2014). Consequently, there is an urgent need to address the limitations of mechanical seed-metering devices during high-speed planting. This crucial advancement in seeding equipment and technology is necessary for improved speed and precision.

A considerable body of research has examined the seed-filling mechanisms and structures of precision seed-metering devices (Li et al., 2023b, 2023; Tang et al., 2022). Du et al. (2019) proposed a method that increased the seed group disturbance to promote seed-filling and investigated the optimal structural parameters of the disturbing strips (Wang et al., 2015a, 2015b, 2017b) improved a pickup finger seed-metering device in order to achieve more precise seeding operations; the authors optimized key components such as the finger clamps, seed clearing zones, and seed guiding belts. Additionally, they investigated the impact of maize size and planting speed on performance. PLANTSYSTEM developed a horizontal disc seed-metering device for maize. The device has gradual holes that efficiently import seeds and remove excess seeds (PLANTSYSTEM Product Information, 2024). While the seeding performance of seed-metering devices has been successfully enhanced in previous research, the applicability of these devices is restricted to low- and medium-speed seeding operations. Under high-speed planting conditions, the seed-filling area of the current seed-metering devices consistently experiences disorder and accumulation of the seed group, mutual extrusion, and constrained posture (Gao et al., 2023; Zhao et al., 2024). Due to seed group extrusion, seeds commonly pile up in front of the seed-taking unit, resulting in empty fillings or multiple simultaneous fillings that cannot be emptied. This often causes no-filling or over-filling problems, resulting in a high-speed filling effect that is not stable. This is a critical problem that needs to be addressed.

To this end, in this research, a novel posture-adjusting seed-metering device was designed for the high-speed planting of maize. This device creates a uniformly distributed seed flow in the seed-filling area by actively adjusting the filling posture of seeds, thus improving the continuous and effective filling probability of the seed-taking unit. This paper presents the design and analysis of the structural parameters of the core component. The influences of the seed posture adjusting efficiency and seed flow distribution characteristics on the seed-filling effect were clarified through EDEM simulation. Finally, the metering performance was optimized and validated by bench testing. These findings provide a reference for improving the high-speed planting capacity of mechanical seed-metering devices.

## 2 Materials and methods

### 2.1 Overall structure and operating principle

**Figure 1:**
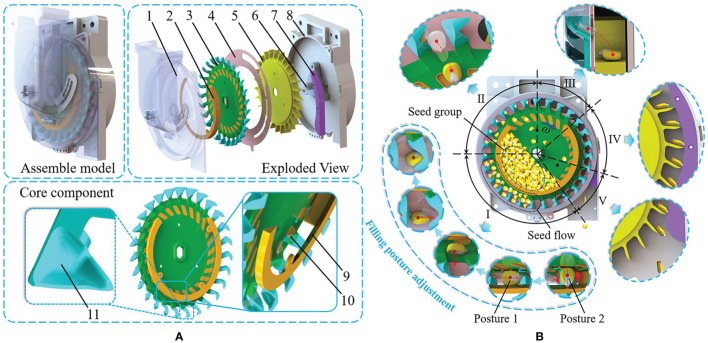
Schematic diagram and operating principle of the seed-metering device. **(A)** Schematic diagram of the seed-metering device. **(B)** Operating principle of the seed-metering device. 1. Front shell 2. Posture-adjusting groove 3. Seeding plate 4. Seed delivery partition 5. Seed guiding wheel 6. Shaft 7. Seed guiding track 8. Rear shell 9. Shrinkage surface 10. Concave surface 11. Seed-taking unit **(I)** Seed-filling II. Seed-clearing III. Seed-delivering IV. Seed-guiding V. Seed-throwing.

**Figure 1A** shows the posture-adjusting precision seed-metering device. The device consist of a front shell, a posture-adjusting groove, a seeding plate, a seed delivery partition, a seed guiding wheel, a seed guiding track, a shaft, and a rear shell. Among them, the seed-taking units are uniformly distributed on the margin of the seeding plate, and concave surfaces are uniformly distributed on the surface of the seeding plate. Together with the posture-adjusting groove, they constitute the posture-adjusting mechanism. This is the core component for adjusting the seed-filling posture, creating a uniformly distributed seed flow in the seed-filling area, and improving the seed-filling effect under high-speed planting conditions.

**Figure 1B** depicts the operating principle of the seed-metering device. The operating principle consists of five consecutive operating processes: seed-filling, seed-clearing, seed-delivering, seed-guiding, and seed-throwing. Seeds enter the seed house and first fall into the posture-adjusting groove of the posture-adjusting mechanism. Due to the shrinkage surface inside the posture-adjusting groove, seeds close to posture 2 (long axis perpendicular to the seeding plate) will be stuck in the groove. When the seeding plate rotates, the concave surface and the posture-adjusting groove will apply a driving torque to the seeds approaching posture 2. This causes the seeds to rotate around the center of mass, adjust to posture 1 (long axis parallel to the seeding plate), and then move with the concave surface to the notch of the posture-adjusting groove in order to drop down into the seed-filling area. Seeds with a consistent posture continuously enter the seed-filling area, forming a uniformly dispersed seed flow driven by the seeding plate. The seeds in the seed flow collide with the seed-taking units and enter them to achieve seed-filling. The remaining seeds in the seed-taking units gradually lose their structural constraints with the turn of the seeding plate and fall to complete seed-clearing. The remaining single seeds that reach the opening of the seed delivery partition slide sideways into the cell at the margin of the seed guiding wheel, achieving seed delivery. Under the guidance of the seed-guiding track, the constrained seeds in the cell are transported along the arc-guiding curve to the seed-throwing port to finish seed-guiding (Dong et al., 2023). Seeds arriving at the seed-throwing port lose their structural constraints and fall vertically with initial velocity and gravity to start the seed-throwing process.

### 2.2 Comparison of seed-filling methods

**Figure 2:**
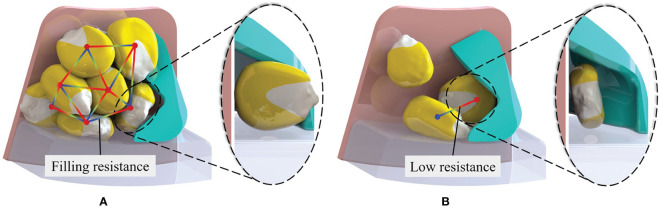
Comparison of seed-filling methods. **(A)** Traditional seed-filling method. **(B)** Novel seed-filling method.

The differences between the seed-filling method developed in this study and traditional seed-filling methods are shown in **Figure 2**. Traditional seed-filling methods use a seed-taking unit structure to extract single seeds directly from the seed group. The filling posture of the seeds is restricted due to the excessive accumulation of seeds in front of the seed-taking unit, resulting in the formation of an inter-constrained force chain (Gao et al., 2019, 2023; Zhang et al., 2024). This situation is exacerbated by an increase in the planting speed, which can lead to the seed-filling posture not matching the seed-taking unit in time, resulting in no-filling (**Figure 2A**). In the novel seed-filling method, the seeds maintain the same postural characteristics (the long axis is parallel to the seed plate) and are detached from the seed group. A moderate quantity of seeds is simply forced in front of the seed-taking unit, with low filling resistance. The seed posture easily matches the seed-taking unit and is not influenced by the planting speed (**Figure 2B**). Therefore, the novel seed-filling method has an increased probability of effective seed-filling and can adapt to higher planting speeds.

### 2.3 Design of the posture-adjusting mechanism

**Figure 3:**
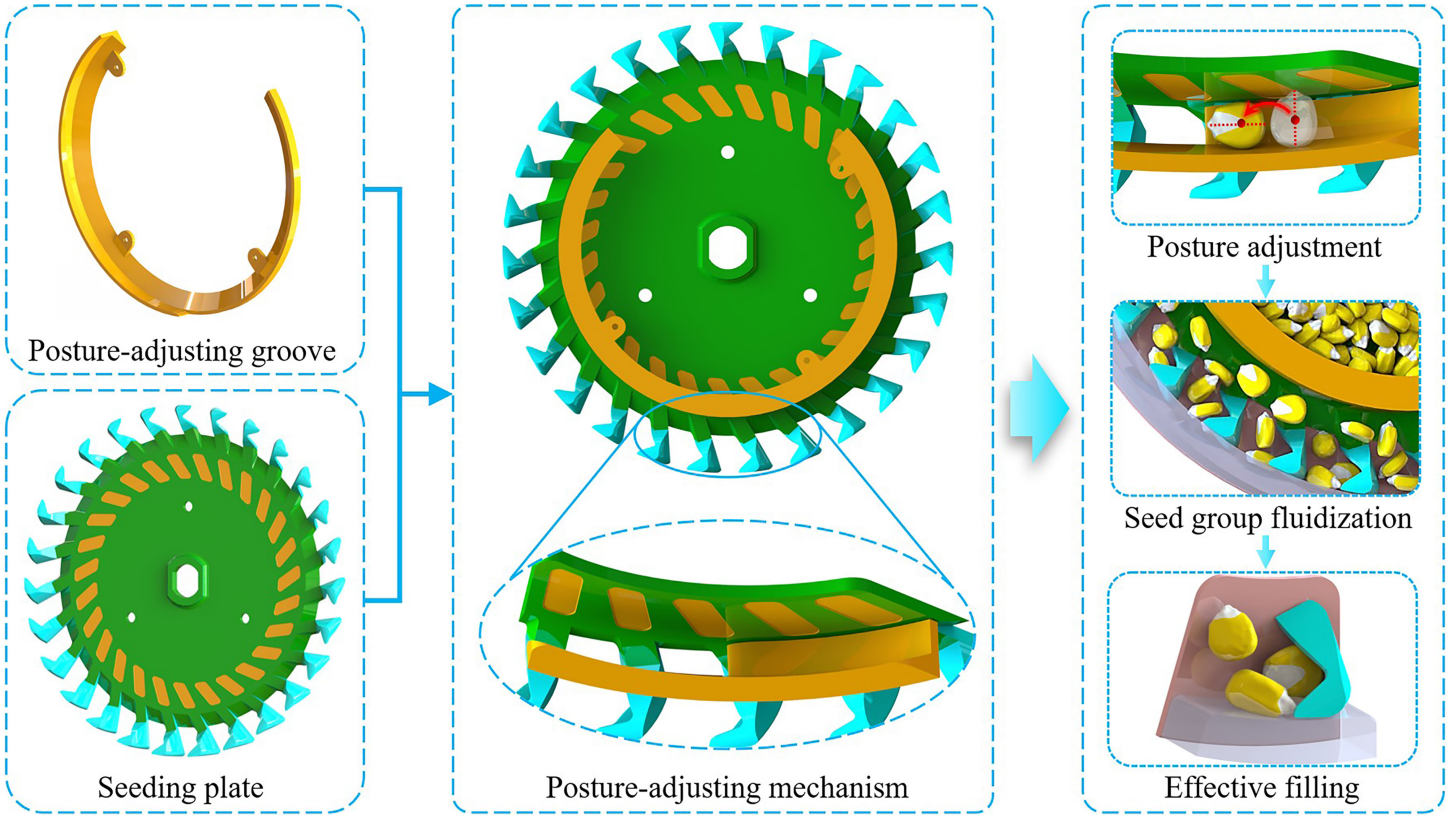
Schematic diagram of posture-adjusting mechanism.

The posture-adjusting mechanism adjusts the posture of the seeds to be filled so that they can break away from the seed group bondage in advance and continuously feed into the seed-filling area to form a uniformly distributed seed flow. Seeds in the seed flow are subjected to simple force in front of the shaped hole and have a low filling resistance, creating highly favorable conditions for effective seed-filling under high-speed conditions. The structural parameters of the posture-adjusting mechanism directly impact the seed-filling effect. The structural parameters primarily compose the seeding plate and the posture-adjusting groove (**Figure 3**).

#### 2.3.1 Design of the seeding plate

**Figure 4:**
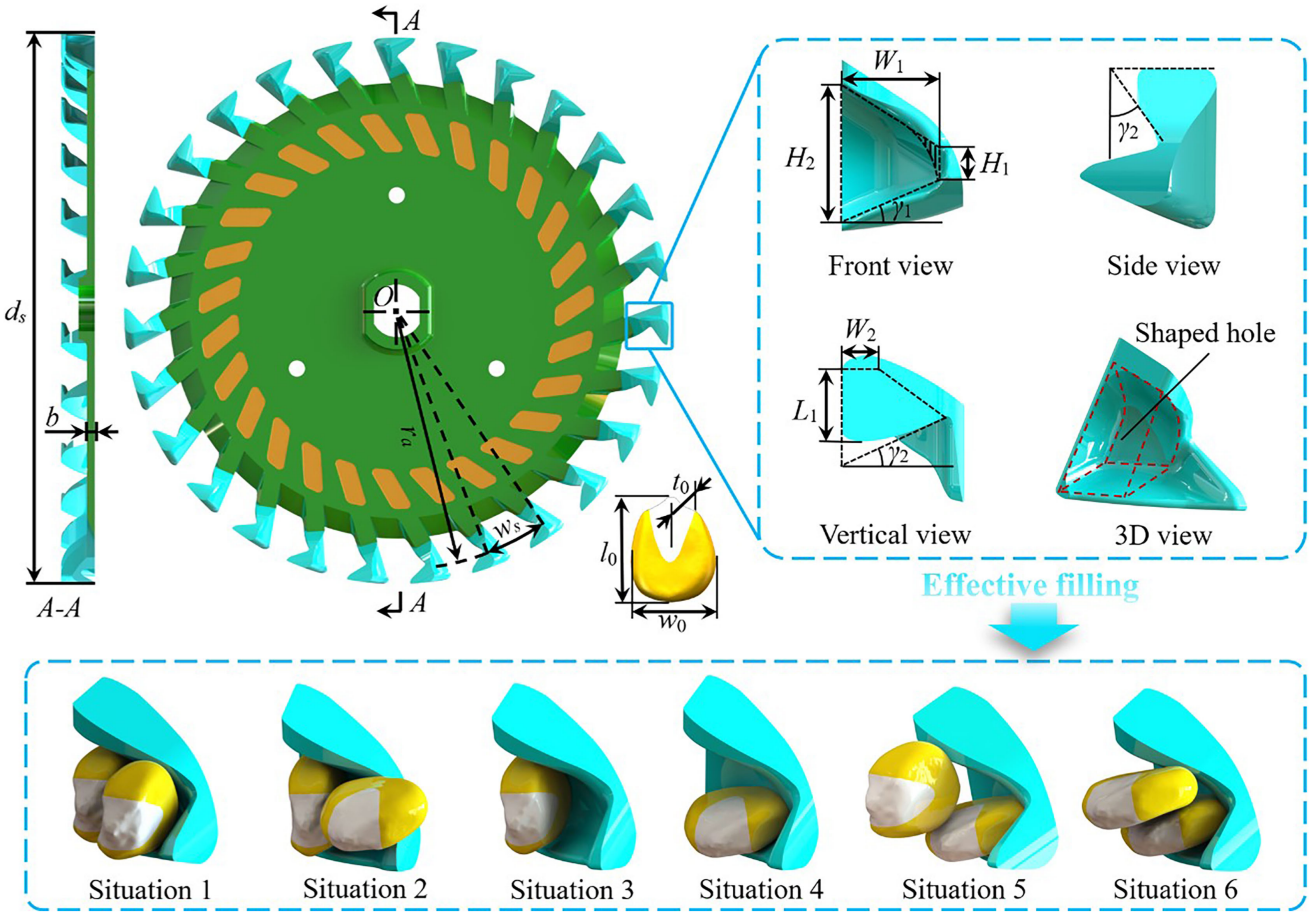
Schematic diagram of seeding plate, seed-taking unit, and effective filling situations.


The number of seed-taking units depends on the diameter of the seeding plate, which is usually 140–260 mm, and should not exceed a reasonable range (Gao et al., 2023). Considering the grain size of maize and the processing requirements, the selected diameter *d_s_* of the seeding plate was 210 mm and the selected thickness *b* was 3 mm. The spacing of the seed-taking unit *w_s_* should be over 1.5 times the maximum seed length of 21.1 mm to prevent the spacing of neighboring seed-taking units from being too small such that seed-filling is hindered (**Figure 4**). Thus, the number of seed-taking units was determined as follows:

(1) $$ws=\frac{2\pi r_{a}}{k}>21.1$$

where *r_a_* is the radius at the center of the seed-taking unit (mm) and *k* is the number of seed-taking units.

It is known that *r_a_* is 97 mm, and substituting this into Equation 1 results in *k* less than 28.9. Accordingly, the number of seed-taking units should be less than 28. To guarantee the reliability of the seed-filling process, the number of seed-taking units selected was 27.

The centrifugal force should be constantly less than the gravity of the seed during seed delivery. From this it follows that:


(2) $$\left\{ \begin{array}{l} n<\frac{30}{\pi }\sqrt{\frac{2g}{10^{-3}d_{s}}}=93.2\\ n=\frac{10000V}{6zk} \end{array}\right.$$

where *n* is the rotational speed of the seeding plate (r/min), *z* is the seeding plant spacing (25 cm), and *V* is the planting speed of the planter (km/h).

From Equation 2, the maximum rotational speed of the seeding plate should not exceed 93.2 r/min. When *k* is taken as 27 and substituted into Equation 2, under planting speeds of 8, 10, 12, and 14 km/h, the corresponding rotational speeds of the seeding plate are 20, 25, 30, and 35 r/min, all of which are far less than 93.2 r/min. Therefore, the device was designed with 27 seed-taking units, meeting the seeding plate design requirements. Further, 27 concave surfaces with a depth of 0.1 mm are distributed on the seeding plate surface to ensure that the seeds can be adjusted to the target posture. These concave surfaces enhance the driving torque required for the seeds during the posture-adjusting process.

#### 2.3.2 Structure parameters of the seed-taking unit

The length *l*, width *w*, and thickness *t* of the maize seeds for this study were based on the published “Zhengdan 958” maize seed measurements, where the average length *l*_0_ of the maize seeds was 11.54 mm, the average width *w*_0_ was 8.63 mm, and the average thickness *t*_0_ was 6.17 mm (Dong et al., 2022).

The seed-taking unit directly impacts the seed posture, shape, and dimension compatibility. The seed-taking unit must have rationally designed structural parameters to effectively fill the seeds (after seed-clearing, one seed remains in the shaped hole). The seed-taking unit’s main structural parameters include the mouth width *W*_1_, the tail width *W*_2_, the wall height *H*_1_, the hole height *H*_2_, the hole length *L*_1_, the bottom surface inclination angle *γ*_1_, and the notch angle *γ*_2_. Their values should be determined based on six specific situations when seeds are effectively filled (**Figure 4**).

The six situations are: two seeds in the side-standing posture are laterally superimposed into the shaped hole (Situation 1); a single seed in the side-standing posture and a single seed in the lying posture are laterally superimposed into the shaped hole (Situation 2); a single seed in side-standing posture into the shaped hole (Situation 3); a single seed in the lying posture into the shaped hole (Situation 4); a single seed in the side-standing posture and a single seed in the lying posture longitudinally superimposed into the shaped hole (Situation 5); two seeds in the lying posture longitudinally superimposed into the shaped hole (Situation 6). It can be deduced that the value of the structural parameters is satisfactory when:


(3) $$\left\{ \begin{array}{l} 0\leq H_{1}-0.5t_{0}\leq 0.5t_{0}\\ 0\leq H_{2}-w_{0}\leq t_{0}\\ 0\leq W_{1}-w_{0}\cos\gamma_{1}\leq t_{0}\cos\gamma_{1}\\ 0\leq W_{2}-0.5t_{0}\leq 0.5t_{0}\\ 0\leq L_{1}\leq l_{0} \end{array}\right.$$

Since the bottom surface inclination angle *γ*_1_ influences the shape of the seed-taking unit’s filling port, the notch angle *γ*_2_ determines the constraint force after the seed filling. When *γ*_1_ is too large, the seed-filling posture cannot match the hole, and when it is too small, many seeds are easily filled into the shaped hole simultaneously. Removing excess seeds is difficult when *γ*_2_ is too large, and when it is too small, all seeds are removed easily. Based on the dimension range of the seeds, the results of the pre-tests, and Equation 3, it was determined that the optimal values of the mouth width *W*_1_, the tail width *W*_2_, the wall height *H*_1_, the hole height *H*_2_, the hole length *L*_1_, the bottom surface inclination angle *γ*_1_, and the notch angle *γ*_2_ were 11 mm, 5 mm, 5 mm, 14 mm, 7 mm, 20°, and 25°, respectively.

#### 2.3.3 Design of the posture-adjusting groove

The posture-adjusting groove is fixed between the front shell and the seeding plate, and a partial notch connects the seed-filling area on the bottom edge. To ensure that seeds close to the target posture (long axis parallel to the seeding plate) can pass through smoothly and the rest of the seeds are blocked, the opening width *b_o_* of the posture-adjusting groove should be close to the seed width. Further, to enable effective seed flow formation in the seed-filling area, the radius of the posture-adjusting groove *r_p_* is 80 mm and the width of the posture-adjusting groove *b_p_* is 13 mm. A 90° opening is present at the top of the posture-adjusting groove to retrieve the cleared-off seeds and to avoid effects on the seed-filling process. The opening angle *θ_o_* of the posture-adjusting groove is the angle between the vertical direction of the center and the opening position. In the pre-test, when the *θ_o_* exceeded 30°, the seed-filling area was insufficient, leading to severe leakage. When it was lower than 0°, the particle density of seed flow was relatively high, inhibiting the seed-filling. Therefore, the opening width and the opening angle were as follows:


(4) $$\left\{ \begin{array}{l} b_{o}\in (7.5,9)\\ \theta_{o}\in (0,30) \end{array}\right.$$

**Figure 5:**
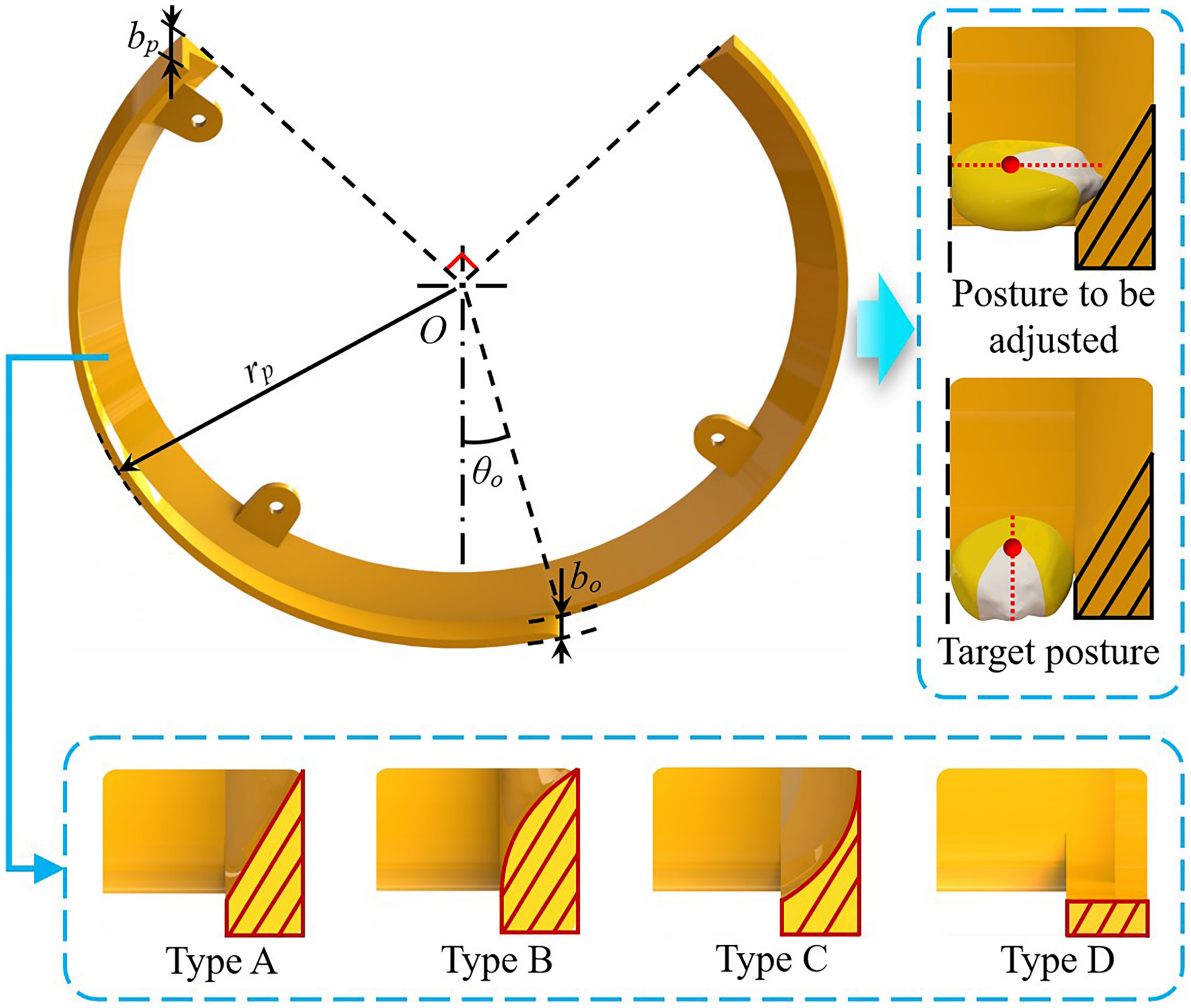
Schematic diagram of posture-adjusting groove.

To ensure that the posture-adjusting mechanism has a favorable adjusting effect on the seed posture, the posture-adjusting groove has a shrinking cross-section to assist the concave surface of the seeding plate to exert sufficient torque on the seeds, prompting their posture to change. Four different structural types were designed for comparison to investigate the influence of the shrinking cross-sections on the posture adjustment and seed-filling effects. Among them, a 60° beveled surface served as type A, projected outward as type B, and recessed inward as type C. Type D, which had no internal structure, was used as a control (**Figure 5**).

#### 2.3.4 Analysis of the seed-filling process

**Figure 6:**
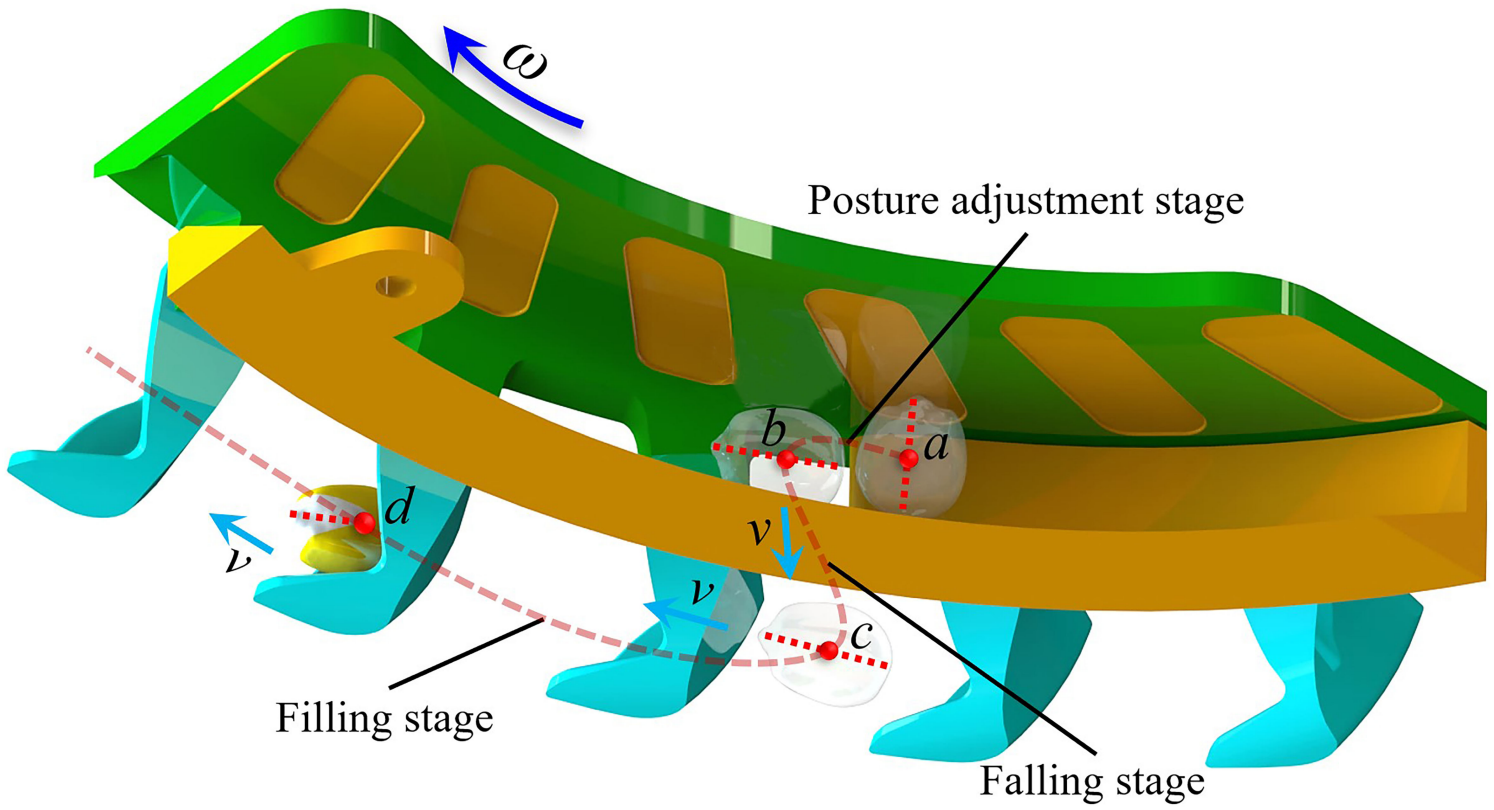
Stage division during the seed-filling process.

The seed-filling process can be divided into several stages based on the seed motion characteristics: the posture adjustment stage, the falling stage, and the filling stage, corresponding to the *ab*, *bc*, and *cd* segments, respectively (**Figure 6**). The main factors influencing the seed-filling effect were clarified by analyzing the seed posture-adjusting efficiency and seed flow distribution characteristics. The normal and tangential contact forces between particles were used to examine the interactions between the seeds, and every stage was analyzed.

**Figure 7:**
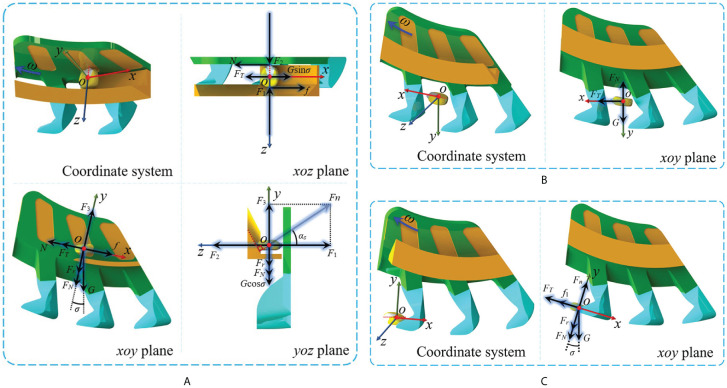
Force analysis during the seed-filling process. **(A)** Posture adjustment stage. **(B)** Falling stage. **(C)** Filling stage.


In the posture adjustment stage, a space coordinate was built with the seed’s center of mass *o* as the base point. The centrifugal force of the seed was contrary to the *y*-axis and vertical to the *x*-axis; the vertical of the seeding plate served as the *z*-axis (**Figure 7A**). The equilibrium equations for the forces on the *xoz*, *xoy*, and *yoz* planes were established as follows:

(5) $$\left\{ \begin{array}{l} N=f=F_{1}\mu =F_{2}\mu \\ N+F_{T}=G\sin\sigma +f\\ F_{3}=F_{r}+F_{N}+G\cos\sigma \\ F_{1}=F_{3}\cot\alpha_{s} \end{array}\right.$$

where *N* is the friction force of the concave surface of the seeding plate on the seed (N), *f* is the friction force of the posture-adjusting groove on the seed (N), *F*_1_ is the *z*-axial force of the seed support force *F_n_* (N), *F*_2_ is the reverse force of *F*_1_ (N), *F*_3_ is the *y*-axial force of the seed support force *F_n_* (N), *μ* is the factor of sliding friction between the mechanism and the seed, *F_N_* is the normal contact force of the seed (N), *F_T_* is the tangential contact force of the seed (N), *G* is the gravity of the seed (N), *F_r_* is the centrifugal force of the seed (N), *σ* is the angle between the gravity and the centrifugal force of the seed (°), and *α_s_* is the angle between the seed support force *F_n_* and reverse direction of the *z*-axis (°).

The seed center of mass was assumed to be at the center of the seed length *l* to simplify the analysis process. The driving torque of the seed was as follows:


(6) $$\Sigma M=\frac{l}{2}(N+f)$$

Based on Equations 5 and 6 can be obtained:


(7) $$\Sigma M=(F_{r}+F_{N}+\sqrt{G^{2}-F_{T}^{2}})\mu l\cot\alpha_{s}$$

Equation 7 shows that the driving torque on the seed is negatively correlated with the angle *α_s_*. Thus, the driving torque during the posture adjustment stage mainly depends on the shape of the shrinking cross-section of the posture-adjusting groove.

In the falling stage, a space coordinate was built with the seed’s center of mass *o* as the origin, the direction of gravity served as the *y*-axis, the vertical direction of gravity as the *x*-axis, and the vertical direction of the seeding plate as the *z*-axis (**Figure 7B**). The force relationship of the seed in the *xoy* plane was as follows:


(8) $$\left\{ \begin{array}{l} F_{x}=F_{T}\\ F_{y}=G-F_{N} \end{array}\right.$$

where *F_x_* is the total force of the seeds on the *x*-axis (N) and *F_y_* is the total force of the seeds on the *y*-axis (N).

From Equation 8, the denser the particles around the seed to be filled in the falling stage, the larger the normal contact force *F_N_* and tangential contact force *F_T_* it receives. The total force of the seed on the *y*-axis direction will be smaller. The efficiency of seed entry into the seed-filling area diminishes, hindering the formation of a uniform seed flow and adversely affecting the seed-filling effect. The sparser the particles around the seed to be filled in the falling stage, the less conducive it is to the formation of a uniform seed flow. A previous study reported that the particle density in the falling stage was positively correlated with the effective seed supply area of the structure (Zong et al., 2023). The opening width *b_o_* and the opening angle *θ_o_* of the posture-adjusting groove determine the size of the effective seed supply area. Thus, it can be inferred that the uniformity of the seed flow in the seed-filling area depends on the *b_o_* and the *θ_o_*.

In the filling stage, a space coordinate was built with the seed center of mass *o* as the origin. The centrifugal force was contrary to the *y*-axis and vertical to the *x*-axis; the vertical of the seeding plate served as the *z*-axis (**Figure 7C**). The force relationship of the seed in the *xoy* plane was as follows:


(9) $$\left\{ \begin{array}{l} F_{x}=G\sin\sigma -F_{T}-f_{1}\\ F_{y}=F_{r}+F_{N}+G\cos\sigma -F_{n}=0\\ f_{1}=\mu F_{n} \end{array}\right.$$

where *f*_1_ is the frictional resistance in the filling stage (N).

From Equation 9:


(10) $$\begin{array}{c} F_{x}=G\sqrt{1+\mu^{2}}\sin(\sigma -\arctan\mu )-\\ \mu F_{r}-\mu F_{N}-F_{T} \end{array}$$

In Equation 10, the seed-filling force is *F_x_*, and this is negatively correlated with its total tangential and normal contact force. When the effective seed supply area of the posture-adjusting groove is too large or too small, there will be a non-uniform distribution of the seed flow. The seeds will be subjected to an unstable tangential and normal contact force, resulting in a filling force that is too large or too small for the seeds. This is not conducive to maintaining a stable seed-filling effect. Since the opening width *b_o_* and the opening angle *θ_o_* determine the size of the effective seed supply area, it can be inferred that *b_o_* and *θ_o_* have direct influences on the seed-filling effect.

Therefore, the seed posture adjusting efficiency and uniformity of the seed flow will influence the seed-filling effect of the seed-metering device under high-speed planting conditions. Low efficiency of seed posture adjustment and unstable seed supply are the main reasons for a non-uniform seed flow distribution, making it easy for no-fill or over-fill to occur. In summary, the seed-filling effect is mainly influenced by the key parameters of the posture-adjusting groove (groove type, opening width *b_o_*, and opening angle *θ_o_*). Thus, these parameters were optimized below in simulations and physical experiments.

### 2.4 Simulation conditions

EDEM2022 discrete element software was chosen to conduct simulation experiments on the key parameters in order to clarify the interactions between the seed flow characteristics and the adjusting mechanism and reveal enhancement mechanism on the seed-filling effect. During the simulation process, Hertz Mindlin (no sliding) was selected as the contact model between the particle and geometry (Chen et al., 2020; Wang et al., 2024). The simulation boundary covered the whole seed-metering device, and the gravity was taken as 9.8 m/s^2^ in the vertical downward direction. The geometry of the seed-metering device was created as igs file format and imported into EDEM software (Liu et al., 2018). The material in the simulation mainly involved the seed-metering device and maize seeds, and the material of the seed-metering device was set as aluminum alloy, according to the real-life processing situation. The physical parameters during the simulation are shown in **Table 1** (Tang et al., 2023; Dong et al., 2024).

**Table 1:** Simulation parameters.

Material	Parameter	Value
Seed particles	Poisson’s ratio	0.4
	Solids density (kg·m^-3^)	1197
	Shear modulus (Pa)	1.37E+08
Seed-metering device (Aluminum alloy)	Poisson’s ratio	0.33
	Solids density (kg·m^-3^)	2700
	Shear modulus (Pa)	2.7E+10
Seed to seed	Coefficient of restitution	0.182
	Coefficient of static friction	0.07
	Coefficient of rolling friction	0.02
Seed to seed-metering device	Coefficient of restitution	0.62
	Coefficient of static friction	0.3
	Coefficient of rolling friction	0.09

**Figure 8:**
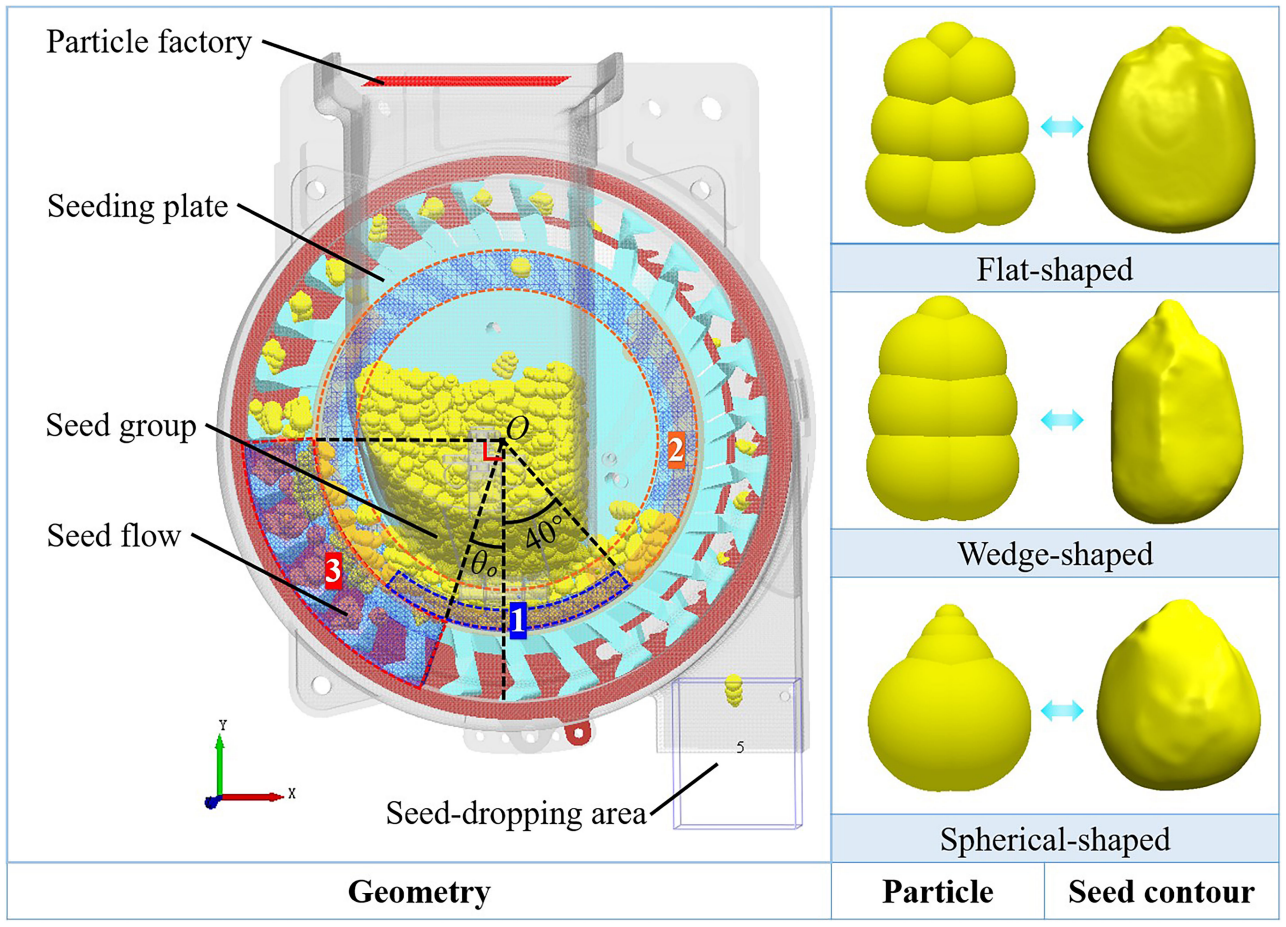
EDEM simulation model.

The particle factory on the seed inlet continuously generated 800 particles for simulation experiments. Among them, the flat-shaped, wedge-shaped, and spherical-shaped particles adopted a spherical splicing model with a quantity ratio of 6: 3: 1. The triaxial dimensions of each type of particle model followed a normal distribution and were close to the actual dimensions (Du and Liu, 2023). The solving process adopted the Euler algorithm, with a fixed time step of 20% and a storage time interval of 0.01 s (**Figure 8**).

In **Figure 8**, to quantitatively analyze the effects of the posture adjustment of particles in the posture-adjusting groove and the seed flow distribution characteristics, three data collection areas, 1–3, were established in the simulation post-processing. Area 1 was arc-shaped, with symmetrical left and right boundaries about the center *O* of the seeding plate, each at a 40° angle. It mainly collected the rotational kinetic energy of the seeds in the posture-adjusting groove. Area 2 was ring-shaped, with its center being the surface center *O* of the seeding plate; it was used to collect torque information from the concave surfaces of the seeding plate. Area 3 was arc-shaped, with the vertical direction of the center *O* of the seeding plate making an angle of *θ_o_* with its right boundary and sitting perpendicular to its left boundary. This area mainly monitored the distribution characteristics of the seed flow in the seed-filling area.

### 2.5 Bench experiment conditions

**Figure 9:**
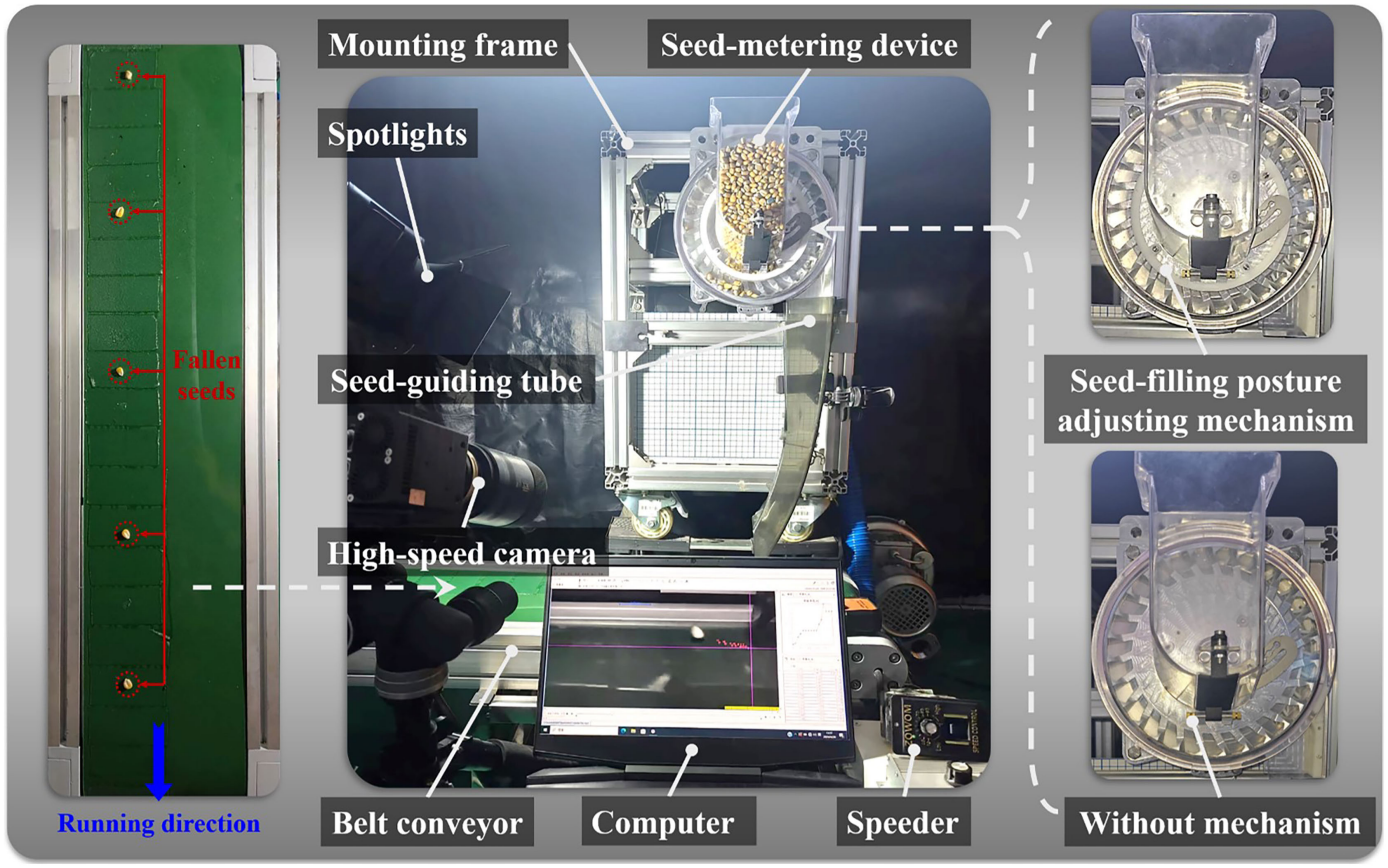
Bench test conditions.

The bench experiment included a performance optimization experiment and a performance validation experiment involving materials such as maize seeds and a posture-adjusting seed-metering device. The main variety of maize seeds was “Zhengdan 958” (thousand seed weight of 290.2 g, moisture content of 10.36%, and rest angle of 22.33°). The critical components of the seed-metering device were machined with 6061 aluminum (CNC processing, accuracy of ±0.01 mm). The test devices were the metering performance test platform, which was self-constructed, and the visual data collection system. The performance test platform consisted of a mounting frame, a drive motor (adjusting speed range 0–35 r/min), a transmission device (input chain, output shaft), a seed-guiding tube (to transport the seeds), and a belt conveyor (speed range 0–14 km/h, with a gelatinized surface to fix the seeds). The visual data collection system consisted of a high-speed camera (Revealer, M230M/C), spotlights, coordinate panels, and motion analysis software (Tracker). The testing conditions are displayed in **Figure 9**.

### 2.6 Experimental design

(1) The type of posture-adjusting groove directly influenced the seed posture-adjusting efficiency, with a more significant effect on the seed-filling effect than the opening angle *θ_o_* and the opening width *b_o_*. Firstly, a simulated comparison experiment was carried out to investigate the effects of the type of posture-adjusting groove on the seed posture-adjusting efficiency, seed flow distribution characteristics, and metering performance. The aim of this was to select the optimal type.

(2) Based on the optimal groove type, a double-factor optimization experiment of the metering performance was carried out using the performance test platform of the seed-metering device (Li et al., 2020). The mathematical relationship model was established between the metering performance indices (pass rate *Y*_1_, repeat rate *Y*_2_, and leak rate *Y*_3_) and experimental factors (opening angle *X*_1_, opening width *X*_2_). The influences of the factors and their interactions on the metering performance were analyzed, and the optimal parameter combination of the seed-metering device was identified.

(3) The validation experiment of the seed-metering device’s high-speed planting capacity was carried out according to its optimal structure parameters. The metering performance at different planting speeds (8 km/h, 10 km/h, 12 km/h, 14 km/h) was tested, respectively, and contrasted with a seed-metering device without a posture-adjusting mechanism.

### 2.7 Experimental methods

The experiment was performed in accordance with the international test method of single seed precision drills in ISO 7256-1 “Sowing equipment**–**Test methods” (Zhao et al., 2024). A maize seeding plant spacing of 0.25 m was selected and 250 segments of seed spacing lengths, seeded in the steady operating condition of the seed-metering device, were selected as the experimental sample. Every group of experiments was repeated three times under the same conditions to ensure the accuracy of the experimental results, and the average was taken as the final statistical result (Li et al., 2021; Zhang et al., 2023). The experimental indices (pass rate *Y*_1_, repeat rate *Y*_2_, and leak rate *Y*_3_) were computed using the visual data collection system, as follows:


(11) $$\left\{ \begin{array}{l} Y_{1}=n_{1}/N^{\prime }\\ Y_{2}=n_{2}/N^{\prime }\\ Y_{3}=n_{3}/N^{\prime } \end{array}\right.$$

where *n*_1_ is the number of pass segments, *n*_2_ is the number of repeat segments, *n*_3_ is the number of leak segments, and *N’* is the number of test samples, i.e., 250.

## 3 Results and discussions

### 3.1 Simulated comparison experiment results and analysis

In the simulated comparison experiment of the different types of posture-adjusting grooves, the opening angle was 20° and the opening width was 8 mm. The relatively higher planting speed requirement of the seed-metering device was met by setting it at 14 km/h (the rotational speed of the seeding plate was 35 r/min).

#### 3.1.1 Analysis of the seed posture-adjusting efficiency

**Figure 10:**
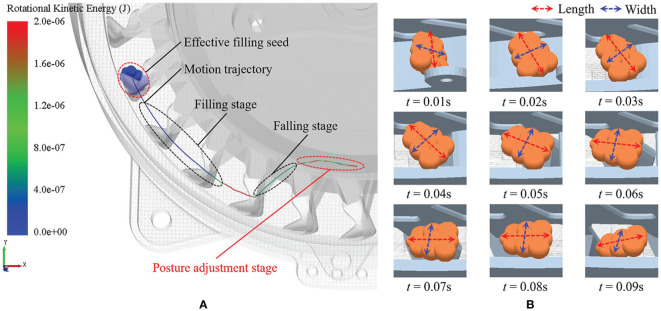
Changes in motion characteristics of single seed during the posture adjustment stage. **(A)** Rotational kinetic energy. **(B)** Seed posture.

**Figure 10A** illustrates the changes in the rotational kinetic energy of the seeds throughout the seed-filling process. In the posture adjustment stage, the change in the rotational kinetic energy of a single seed was more significant than in the falling and filling stages. This is because the driving torque constantly changed the seed posture during the posture adjustment stage, resulting in significant fluctuation in the rotational kinetic energy as compared to the other stages.

**Figure 10B** depicts the specific changes from 0.01–0.09 s in the posture of a single seed during the posture adjustment stage in the simulation. The seed posture with the long axis perpendicular to the seeding plate was continuously adjusted until it was parallel to the seeding plate and then ready to fall, achieving the posture-adjusting effect.

**Figure 11:**
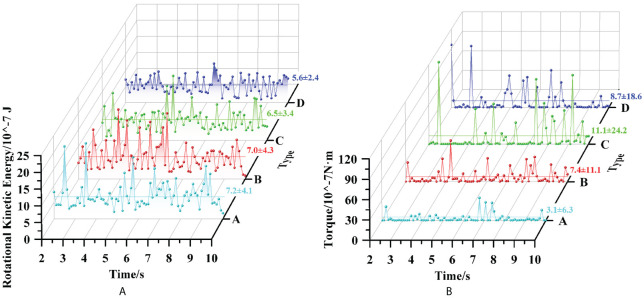
The influence of groove types on the posture adjusting efficiency. **(A)** Rotational kinetic energy of seed group. **(B)** Torque of concave surfaces.

**Figure 11A** displays the influences of different types of posture-adjusting grooves on the rotational kinetic energy of the seed group over 2–10 s of the simulation. Type A corresponded to rotational kinetic energy between 1.7×10^-7^–22.7×10^-7^ J, with an average of 7.2×10^-7^ J. Type B corresponded to rotational kinetic energy between 1.1×10^-7^–21.2×10^-7^ J, with an average of 7.0×10^-7^ J. Type C corresponded to rotational kinetic energy ranging from 1.3×10^-7^ to 19.8×10^-7^ J, with an average of 6.5×10^-7^ J. Type D corresponded to rotational kinetic energy ranging from 1.0×10^-7^ to 12.1×10^-7^ J, with an average of 5.6×10^-7^ J.

**Figure 11B** displays the influences of different types of posture-adjusting grooves on the torque in the concave surfaces of the seeding plate over 2–10 s of the simulation. Type A corresponded to a torque between 0–33×10^-7^ Nm, with an average torque of 3.1×10^-7^ Nm. Type B corresponded to a torque between 0.1–62×10^-7^ Nm, with an average of 7.4×10^-7^ Nm. Type C corresponded to a torque between 0–124.5×10^-7^ Nm, with an average of 11.1×10^-7^ Nm. Type D corresponded to a torque between 0–96.5×10^-7^ Nm, with an average of 8.7×10^-7^ Nm.

The comparison indicated that type A had a greater effect on increasing the rotational kinetic energy of the seeds, with this being significantly higher than type D. Simultaneously, type A corresponded to the lowest torque of the concave surfaces of the seeding plate, being significantly lower than types C and D. This is because the driving torque of the seeds in the type A posture-adjusting groove was more consistent than that of the other types. The seeds were not subjected to excessive or insufficient torque, which ensuring a higher efficiency in seed posture adjustment.

#### 3.1.2 Analysis of seed flow distribution characteristics

**Figure 12:**
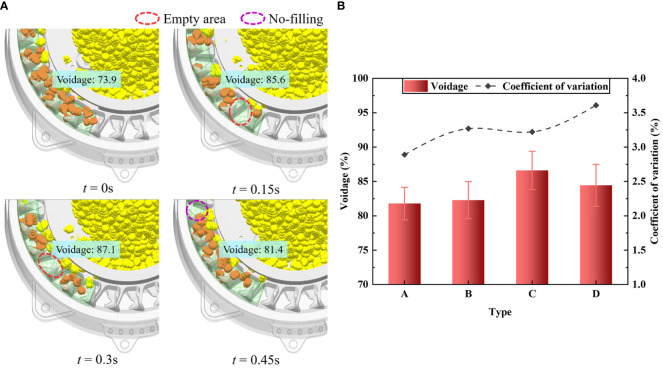
Distribution characteristic of seed flow in the seed-filling area. **(A)** The influence of voidage on seed-filling effect. **(B)** The influence of groove type on seed flow characteristics.

**Figure 12A** illustrates the seed flow distribution in the seed-filling area from 0–0.45 s in the simulation. When *t* was 0 s, the seed flow distribution was relatively uniform, with a voidage of 73.9%. When *t* was 0.15 s, the seed flow had a clear empty area, and the voidage increased rapidly to 85.6%. When *t* was 0.3 s, the position of the empty area in the seed flow increased with the turn of the seeding plate, and the voidage was 87.1%. When *t* was 0.45 s, the empty area was not replenished with seeds in time, resulting in the seed-taking unit not being filled; the voidage was 81.4%. This analysis demonstrated that a non-uniform distribution of the seed flow due to an empty area is the main cause of the leakage phenomenon.

**Figure 12B** displays the average values and coefficients of the variation in seed flow voidage in the seed-filling area under different types of posture-adjusting grooves within 2–10 s of the simulation. Type A corresponded to an average value of 81.8%; the coefficient of variation was the smallest at 2.9%. Type D corresponded to an average value of 84.4%; the coefficient of variation was the largest at 3.6%. Under type B and C conditions, the coefficients of variation between 2.9% to 3.6%. These results indicate that the fluctuation in the seed flow voidage was the least under type A, and the seeds were more uniformly distributed, with a lower chance of having empty areas, as compared to the other types.

#### 3.1.3 Simulation validation

**Figure 13:**
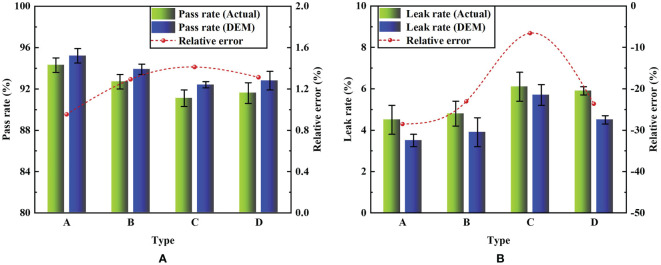
Comparison of experiment results between actual and simulation. **(A)** Comparison of pass rate. **(B)** Comparison of leak rate.

**Figure 13** presents the simulation and actual comparison of the metering performance under different posture-adjusting grooves. Type A exhibited the optimal metering performance compared with the other types. Under this condition, the simulated pass rate was 95.2% and the actual pass rate was 94.3%. The pass rate was the highest compared with the other types. The simulated leak rate was 3.5% and the actual leak rate was 4.5%. The leak rate was the lowest of the four types. These findings indicate that type A significantly improved the metering performance compared to the other types. The relative errors of the pass rate between the simulation and the actual experiment were between 1.0% and 1.4%, and the relative errors of the leak rate were between -28.6% and -6.6%. These are within reasonable ranges, indicating that the simulation was relatively accurate.

It can be deduced that when the shrinking cross-section of the posture-adjusting groove is curved or has no shape, the driving torque of the seeds at different positions in the groove is inconsistent. This is not conducive to the timely adjustment of the seed posture into the seed-filling area. In addition, the seed particle model in the simulation cannot reflect the differences in the actual seed shape. In the actual experiment, there are processing and assembly errors in the seed-metering device, and the transmission system will produce vibration and other physical factors. The above factors will cause errors between the actual and simulation indices, but these errors are within a reasonable range and have no influence on the experimental results. Therefore, based on the above, further experiments on the metering performance of the device were carried out using the type A posture-adjusting groove.

### 3.2 Double-factor experimental results and analysis

#### 3.2.1 Analysis of the experiment results

Based on the results of the simulated comparison experiment, a double-factor optimization experiment was performed with a planting speed of 14 km/h. **Table 2** displays the differences between the factor levels in the experimental results (data presented as the average ± standard deviation).

**Table 2:** Program and results of the double-factor experiment.

No.	Factor		Index		
	*X*_1_ (°)	*X*_2_ (mm)	*Y*_1_ (%)	*Y*_2_ (%)	*Y*_3_ (%)
1	0	7.5	91.1 ± 1.0aA	1.5 ± 0.2aB	7.4 ± 1.1cA
2	0	8	92.8 ± 1.0abAB	1.9 ± 0.5abB	5.3 ± 0.5bA
3	0	8.5	94.9 ± 0.7cA	2.7 ± 0.5bB	2.4 ± 0.3aA
4	0	9	93.5 ± 0.5bcA	3.9 ± 0.5cB	2.6 ± 0.5aA
5	10	7.5	90.0 ± 0.7aA	1.3 ± 0.2aB	8.7 ± 0.8cAB
6	10	8	91.8 ± 0.7bA	1.3 ± 0.5aAB	6.9 ± 0.4bB
7	10	8.5	94.4 ± 0.9cA	1.6 ± 0.3aA	4.0 ± 0.6aB
8	10	9	93.3 ± 0.5cA	2.8 ± 0.7bAB	3.9 ± 0.7aAB
9	20	7.5	90.7 ± 0.5aA	0.1 ± 0.2aA	9.2 ± 0.7bAB
10	20	8	94.7 ± 0.7cC	0.8 ± 0.3aA	4.5 ± 0.5aA
11	20	8.5	94.8 ± 0.3cA	1.6 ± 0.3bA	3.6 ± 0.3aAB
12	20	9	93.4 ± 0.5bA	2.5 ± 0.4cA	4.1 ± 0.8aAB
13	30	7.5	89.7 ± 0.5aA	0.3 ± 0.2aA	10.0 ± 0.7bB
14	30	8	94.2 ± 0.7bBC	0.5 ± 0.2aA	5.3 ± 0.5aA
15	30	8.5	93.4 ± 0.7bA	1.3 ± 0.2bA	5.3 ± 0.8aC
16	30	9	92.8 ± 0.7bA	2.1 ± 0.5cA	5.1 ± 0.5aB

Different lowercase letters indicate significant differences between indices at different *X*_2_ levels under the same *X*_1_ level. Different capital letters indicate significant differences between indices at different *X*_1_ levels under the same *X*_2_ level.

The effect of the opening angle on the pass rate was insignificant, and the effect of the opening width on the pass rate was significant. When the opening angle was fixed, the pass rate showed a rising and then falling trend with increases in the opening width. The pass rate was higher when the opening width was between 8 mm and 8.5 mm. When the opening width was fixed, the pass rate fluctuated slightly with increases in the opening angle. The pass rate was relatively high when the opening angle was 20°. The effects of both the opening angle and opening width on the repeat rate were significant. When the opening angle was fixed, the repeat rate increased with increases in the opening width and was smallest at an opening width of 7.5 mm. When the opening width was fixed, the repeat rate decreased with increases in the opening angle and was smallest with an opening width of 30°. The effect of the opening angle on the leak rate was insignificant, and the effect of the opening width on the leak rate was significant. When the opening angle was fixed, the leak rate tended to decline with increases in the opening width; it was relatively low when the opening width was between 8 mm and 9 mm. When the opening width was fixed, the leak rate slowly increased with increases in the opening angle. The leak rate was relatively low when the opening angle was between 0° and 20°.

This may be because the distribution characteristics of the seed flow are determined by both the opening angle and the opening width. When the opening angle is too small or too large, the seed flow distribution area will be too scattered or concentrated, resulting in a non-uniform distribution of the seed flow or insufficient seed-filling time. When the opening width is too small or too large, the particle density of the seed flow will be too low or too high, and the seed-taking unit will not be able to capture the seeds promptly or suppress the seed-filling effect. These two factors will exacerbate multiple or leakage seeding, thereby influencing the high-speed planting capacity of the seed-metering device.

#### 3.2.2 Regression analysis

Multiple linear regression analysis was performed on the experimental results in **Table 3** using Design Expert 8.0.6 software to examine the significant effects of the different experimental factors, such as the opening angle *X*_1_ and opening width *X*_2_, and their interaction effects on the pass rate *Y*_1_, repeat rate *Y*_2_, and leak rate *Y*_3_ metering performance indices (**Table 3**).

**Table 3:** Significance analysis of experimental factors on indexes.

Source	*Y*_1_	*Y*_2_	*Y*_3_
Model	0.0018 **	< 0.0001 **	< 0.0001 **
*X*_1_	0.7298	0.0010 **	0.1549
*X*_2_	0.0017 **	< 0.0001 **	< 0.0001 **
*X*_1_ *X*_2_	0.7151	0.6386	0.6238
*X*_1_^2^	0.8484	0.0360	0.6788
*X*_2_^2^	0.0009 **	0.0116 *	0.0030 **

* means significant, and ** means highly significant.

From **Table 3**, it can be seen that the effects of the opening angle on the pass rate and leak rate were not significant, but the effect on the repeat rate was highly significant. The effects of the opening width on the pass rate, repeat rate, and leak rate were all highly significant. The interaction between the experimental factors had no significant effect on each experimental index. Based on the significance of the analysis results, a multiple regression equation between the experimental factors and indices was established, as follows:


(12) $$\left\{ \begin{array}{l} Y_{1}=-204.1+0.1X_{1}+70.2X_{2}-0.01X_{1}X_{2}-0.0004X_{1}^{2}-4.1X_{2}^{2}\\ Y_{2}=38.2-0.05X_{1}-10.1X_{2}-0.004X_{1}X_{2}+0.001X_{1}^{2}+0.7X_{2}^{2}\\ Y_{3}=265.9-0.06X_{1}-60.1X_{2}+0.02X_{1}X_{2}-0.0009X_{1}^{2}+3.4X_{2}^{2} \end{array}\right.$$

In order to identify the optimal combination of structural parameters for the seed-metering device, the regression equation was solved with the optimization objective of maximizing the pass rate, minimizing the repeat rate, and minimizing the leak rate (Jia et al., 2018; Ding et al., 2020). The objective function and constraints were as follows:


(13) $$\left\{ \begin{array}{l} \max Y_{1}(X_{1},X_{2})\\ \min Y_{2}(X_{1},X_{2})\\ \min Y_{3}(X_{1},X_{2})\\ \text{s.t.}\{\begin{array}{l} 0\leq X_{1}\leq 30{}^{\circ}\\ 7.5\text{mm}\leq X_{2}\leq 9\text{mm} \end{array} \end{array}\right.$$

The results indicated that when the opening angle was 21.6° and the opening width was 8.4 mm, the high-speed planting capacity of the seed-metering device was optimum. Under this condition, the pass rate reached 94.3%, the repeat rate reached 1.2%, and the leak rate reached 4.5%.

### 3.3 Performance validation experimental results and analysis

Based on the optimal combination of structural parameters, the metering performance of the posture-adjusting seed-metering device was tested at different planting speeds, i.e., 8, 10, 12, and 14 km/h, with an opening angle of 21.6° and an opening width of 8.4 mm. It was then compared with a seed-metering device without a posture-adjusting mechanism.

**Figure 14:**
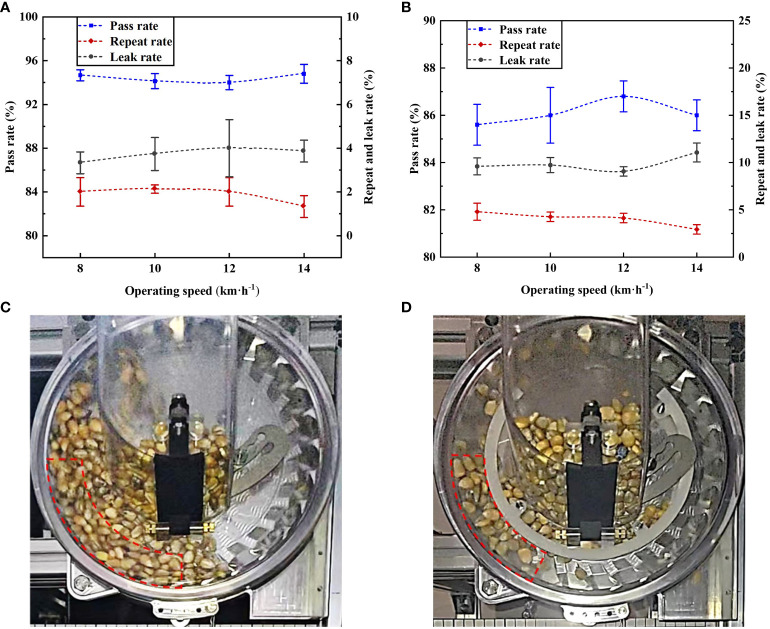
Results of performance validation experiment. **(A)** The posture-adjusting seed-metering device. **(B)** The seed-metering device without posture-adjusting mechanism. **(C)** Seed group. **(D)** Seed flow.

**Figure 14A** demonstrates that the posture-adjusting seed-metering device had a pass rate above 94%, a repeat rate below 2.1%, and a leak rate below 3.9% within the planting speed range of 8–14 km/h. This meets the technical requirements for precision seeding. The pass rate did not significantly change with increases in the planting speed. It remained in the range of 94–95%, indicating that the posture-adjusting seed-metering device has good adaptability to high-speed planting. The analysis indicated that since the seed posture was regulated in advance and the seeds were present in the seed-filling area, there was a uniformly distributed seed flow. This effectively avoided the extrusion effect between seeds, greatly increased the probability of effective seed-filling, and created favorable conditions for maintaining consistent plant spacing and an even distribution of maize under actual high-speed and high-density planting conditions. In particular, the metering performance at a speed of 14 km/h was optimal, with a pass rate of 94.8%, a repeat rate of 1.3%, and a leak rate of 3.9%; the error was small compared to the optimization results. Therefore, the optimal structural parameters of the seed-metering device are as follows: opening angle of 21.6°, opening width of 8.4 mm.

**Figure 14B** demonstrates that the posture-adjusting seed-metering device had significantly improved metering performance compared to the seed-metering device without a posture-adjusting mechanism within the planting speed range of 8–14 km/h. The pass rate of the posture-adjusting seed-metering device was 7.2–9.1% greater than that of the seed-metering device without a posture-adjusting mechanism. When the seed-metering device does not have a posture-adjusting mechanism, due to the significant accumulation of the seed group in the seed-filling area, the seed posture is constrained; thus, this seed-filling method cannot adapt to high-speed planting conditions (**Figure 14C**). However, the seed-metering device with a posture-adjusting mechanism can adjust the seed-filling posture in advance. It creates a uniform seed flow in the seed-filling area, significantly increasing the probability of effective seed-filling under high-speed planting conditions (**Figure 14D**). Together, these findings indicate that the seed-metering device with a posture-adjusting mechanism has an improved high-speed planting capacity compared to traditional methods and is effective under high-speed planting conditions.

### 3.4 Discussion

In previous studies, when the planting speed was increased from 8–14 km/h, the pass rate of a picker finger seed-metering device decreased from 87.1% to 82%, and the pass rate of a spoon wheel seed-metering device decreased from 90.3% to 81.6% (Dong et al., 2022, 2024). Here, the pass rate of the posture-adjusting seed-metering device was 7.7 to 13.1% higher than an existing mechanical seed-metering device at the same planting speed of 8–14 km/h. This indicates that the device described here has better adaptability to high-speed planting conditions and can meet higher technical requirements. This is primarily because when existing mechanical seed-metering devices are planted under high-speed conditions, seed accumulation and extrusion in the seed-filling area and posture restrictions are aggravated, making it difficult to realize effective seed-filling. This leads to heavy leakage seeding and a decline in seeding quality. In this study, the seed-metering device changed the seed-filling posture in advance through the posture-adjusting mechanism. This resulted in a uniformly distributed seed flow in the seed-filling area, which created favorable conditions for continuous and effective seed-filling and significantly improved the device’s adaptability to high-speed planting conditions.

## 4 Conclusions

In order to increase the capability of maize mechanical seed-metering devices for high-speed planting, it was proposed that the seed-filling posture be adjusted and fluidizing of the seed group be performed. To this end, a novel posture-adjusting seed-metering device was designed. The influences of the seed posture adjustment efficiency and seed flow distribution characteristics on the seed-filling effect were clarified. The key structural parameters were optimized and validated. The conclusions are as follows:

(1) The simulated comparison experiment showed that when the posture-adjusting groove was type A, the efficiency of the seed posture adjustment was the highest, the driving torque in the concave surfaces of the seeding plate was the smallest, and the seed flow distribution was more uniform, resulting in a significant improvement in the seed-filling effect.

(2) The double-factor optimization experiment showed that when the opening angle was 21.6° and the opening width was 8.4 mm, the high-speed planting capacity of the seed-metering device was optimal. After validation, it was found that the pass rate, repeat rate, and leak rate were 94.8%, 1.3%, and 3.9%, respectively, under this condition.

(3) The performance validation experiment showed that the posture-adjusting seed-metering device had a pass rate above 94%, a repeat rate below 2.1%, and a leak rate below 3.9% within the planting speed range of 8–14 km/h. It exhibited significant improvement compared to a seed-metering device without a posture-adjusting mechanism. The increase in the pass rate reached 7.2 to 9.1% within the planting speed range of 8–14 km/h. These findings indicate that seed posture-adjusting technology can effectively improve the high-speed planting capacity of mechanical seed-metering devices.

## Data Availability

The original contributions presented in the study are included in the article/supplementary material. Further inquiries can be directed to the corresponding author.
